# Loss of Melanopsin-Expressing Ganglion Cell Subtypes and Dendritic Degeneration in the Aging Human Retina

**DOI:** 10.3389/fnagi.2017.00079

**Published:** 2017-04-04

**Authors:** Gema Esquiva, Pedro Lax, Juan J. Pérez-Santonja, José M. García-Fernández, Nicolás Cuenca

**Affiliations:** ^1^Department of Physiology, Genetics and Microbiology, University of AlicanteAlicante, Spain; ^2^Alicante Institute for Health and Biomedical Research (ISABIAL-FISABIO Foundation)Alicante, Spain; ^3^Department of Ophthalmology, Alicante University General HospitalAlicante, Spain; ^4^Department of Morphology and Cellular Biology, Institute of Neuroscience Principado de Asturias (INEUROPA), University of OviedoOviedo, Spain; ^5^Institute Ramón Margalef, University of AlicanteAlicante, Spain

**Keywords:** retina, melanopsin ganglion cells, circadian rhythms, immunohistochemistry, retinal cell mosaics

## Abstract

In mammals, melanopsin-expressing retinal ganglion cells (mRGCs) are, among other things, involved in several non-image-forming visual functions, including light entrainment of circadian rhythms. Considering the profound impact of aging on visual function and ophthalmic diseases, here we evaluate changes in mRGCs throughout the life span in humans. In 24 post-mortem retinas from anonymous human donors aged 10–81 years, we assessed the distribution, number and morphology of mRGCs by immunostaining vertical retinal sections and whole-mount retinas with antibodies against melanopsin. Human retinas showed melanopsin immunoreactivity in the cell body, axon and dendrites of a subset of ganglion cells at all ages tested. Nearly half of the mRGCs (51%) were located within the ganglion cell layer (GCL), and stratified in the outer (M1, 12%) or inner (M2, 16%) margin of the inner plexiform layer (IPL) or in both plexuses (M3, 23%). M1 and M2 cells conformed fairly irregular mosaics, while M3 cell distribution was slightly more regular. The rest of the mRGCs were more regularly arranged in the inner nuclear layer (INL) and stratified in the outer margin of the IPL (M1d, 49%). The quantity of each cell type decrease after age 70, when the total number of mRGCs was 31% lower than in donors aged 30–50 years. Moreover, in retinas with an age greater than 50 years, mRGCs evidenced a decrease in the dendritic area that was both progressive and age-dependent, as well as fewer branch points and terminal neurite tips per cell and a smaller Sholl area. After 70 years of age, the distribution profile of the mRGCs was closer to a random pattern than was observed in younger retinas. We conclude that advanced age is associated with a loss in density and dendritic arborization of the mRGCs in human retinas, possibly accounting for the more frequent occurrence of circadian rhythm disorders in elderly persons.

## Introduction

Melanopsin is an opsin protein identified in a third class of ocular photoreceptors that are neither rods nor cones (Berson et al., 2002; Hannibal et al., 2002; Hattar et al., 2002; Panda et al., 2002). In mammals, it is expressed in an intrinsically photosensitive subset of retinal ganglion cells (ipRGCs) that produce light-induced responses without the requisite of receiving synaptic input from other neurons in the retina (Provencio et al., 2000; Berson et al., 2002). Melanopsin-expressing retinal ganglion cells (mRGCs) participate in circadian and non-visual light-induced responses, which include the photic entrainment of the circadian system, pupillary light reflex (PLR), post-illumination pupil response (PIPR) and masking responses to light (Panda et al., 2002; Güler et al., 2008). The ipRGCs thus project to a number of brain areas whose functions are not associated with image formation, such as the suprachiasmatic nucleus (SCN) in the case of circadian photoentrainment (Hattar et al., 2003) and the olivary pretectal nucleus (OPN), which controls pupillary reflex (Hattar et al., 2002, 2003). Recently, it has been demonstrated that ipRGCs are implicated directly in cognitive and mood functions by projecting to limbic regions, as the lateral habenula and the medial amygdala (LeGates et al., 2012). In primate retinas, ipRGCs are known to communicate color and irradiance, and to project to the lateral geniculate nucleus (LGN; Berson, 2003; Dacey et al., 2005), contributing to image-forming processes. More recently, it has been reported that melanopsin is also present in the human retina in the outer segments of a cone population that do not appear to express any other opsins. The expression of melanopsin by these cones and their contribution to the conscious perception of light in humans supports the idea that this particular opsin can also act as a classical photopigment (Dkhissi-Benyahya et al., 2006).

In rodents, five subtypes of ipRGCs have been identified (M1–M5; Ecker et al., 2010; Esquiva et al., 2013; Reifler et al., 2015), whereas two subtypes of monostratified ipRGCs have been reported in both human and primates, one sending dendrites to the outer stratum of the inner plexiform layer (IPL) and the second stratified in the IPL inner stratum (Hannibal et al., 2004, 2014; Dacey et al., 2005; Liao et al., 2016). Labeled somas can be found in both the inner nuclear layer (INL) and the ganglion cell layer (GCL), respectively. More recently, it has been described in primates that some ipRGC dendrites cross the IPL to ramify in the opposite stratum (Liao et al., 2016).

Pupillometric measurements indicate that ipRGC function is compromised in diseases of both the optic nerve and the retina, including glaucoma (Feigl et al., 2011), diabetic retinopathy (Feigl et al., 2012), retinitis pigmentosa (Kawasaki et al., 2012), Leber’s hereditary optic neuropathy (Kawasaki et al., 2010), and age-related macular degeneration (Maynard et al., 2015). On the other hand, it has been described that aging affects retinal functions, including electroretinographic responses (Jackson et al., 2002), sensitivity to contrast (Elliott et al., 1990), visual field sensitivity (Johnson et al., 1989; Spry and Johnson, 2001) and adaptation to darkness (Jackson et al., 1999). Furthermore, a large body of literature links aging to sleep alterations. These changes may be the result of abnormal homeostatic sleep regulation, and/or a dysfunction affecting the regulation of circadian rhythms (Cajochen et al., 2006). Circadian rhythms in elderly subjects have been reported to exhibit progressively decreased amplitude, phase advancement and shorter periods (Myers and Badia, 1995; Cajochen et al., 2006).

Multiple factors have been associated with the disturbances in circadian timing that occur with age (Van Someren, 2000; Karasek and Reiter, 2002). These include many degenerative changes which have been identified in the retina and optic nerve of aging people (Johnson et al., 1987; Gao and Hollyfield, 1992; Curcio and Drucker, 1993; Harman et al., 2000; Eliasieh et al., 2007). However, measurements of PIPRs have shown no effects of aging on ipRGC inputs to the pathway controlling the pupil in either humans (Adhikari et al., 2015) or rodless/coneless (rd/rd cl) mice (Semo et al., 2003). Furthermore, several authors have found data supporting the existence of a highly efficient system for ipRGCs survival (Vugler et al., 2008; Cui et al., 2015; Nadal-Nicolás et al., 2015), as these cells show high resistance to injury, even in advanced stages of retinal degeneration (Esquiva et al., 2013; Cuenca et al., 2014; García-Ayuso et al., 2015; Lax et al., 2016).

Our objective was to establish whether aging-related circadian disruptions in humans are associated with changes in ipRGCs. To accomplish this, in the present study we analyze in detail the cellular diversity of mRGCs in the human retina, and assess possible variations in the number, morphology and distribution of these cells in the retinas of 24 human donors, aged 10–81 years.

## Materials and Methods

### Human Retinas

Twenty-four human cadaver eyes were obtained from the eye bank of the University General Hospital of Alicante, Spain, within 6 h of death. They did not report any personal or familial history of retinal dystrophy. All donors signed a consent, which includes that if the organs are not used for transplant, they can be used for research. The human eye donors, both men and women, ranged in age from 10 to 81 years at death, and had no past history of ocular diseases. All subjects gave written informed consent in accordance with the Declaration of Helsinki. This study was carried out in accordance with the recommendations and protocols approved by the Ethics Committee of the University of Alicante.

### Retinal Histology

The human eyeballs were fixed in paraformaldehyde (4% w/v) for 2 h at room temperature (RT), washed in PBS and then cryoprotected in 15%, 20% and 30% sucrose. The lens and vitreous body were removed, and the retina was spread out and flattened, producing four portions: the superior, inferior, nasal and temporal. The macula was identified according to its pigmentation and the pattern of blood vessels in the retina. In each retina, a representative region of around 1 cm^2^ from the superior-nasal area was processed for vertical sections or flat mount. One entire 56-year-old flat-mounted human retina was processed and analyzed to provide a full perspective of the number and distribution of mRGCs.

### Antibodies

Immunohistochemistry techniques were performed using an anti-melanopsin antibody (Provencio et al., 2000; rabbit polyclonal antibody, UF028, 1:5000 dilution) raised against the 15 N-terminal amino acids of human melanopsin, which was supplied by Dr. Ignacio Provencio (University of Virginia, Charlottesville, VA, USA). Biotinylated goat anti-rabbit IgG (1:100, 111-064-144; Jackson ImmunoResearch Laboratories, West Grove, PA, USA) was used as secondary antibody for immunoperoxidase labeling.

### Immunoperoxidase Labeling

Vertical sections and retinal flat mounts were processed using the immunoperoxidase technique, according to the procedures already reported in previous works in great detail (Esquiva et al., 2013). Briefly, after suppression of endogenous hydrogen peroxide (H1009; Sigma, St. Louis, MO, USA), permeability was enhanced by preliminary incubation in 2.28% sodium metaperiodate (S1878; Sigma), followed by incubation in 0.02% sodium borohydride (163314; Panreac, Barcelona, Spain). After a blocking step, retinas were incubated in the primary antibody for 4 days, washed and then incubated in the biotinylated secondary antibody for 3 days. They were subsequently washed, and incubated for two more days in avidin-biotin complex (ABC; PK-6100, Vectastain Elite ABC Kit; Vector Laboratories Ltd., Cambridgeshire, UK). Finally, retinas were washed and incubated with 3,3′-diaminobenzidine tetrahydrochloride (DAB, D5637; Sigma) containing 0.01% H_2_O_2_ and 0.025% ammonium nickel (II) sulfate hexahydrate (A1827; Sigma). Flat retinas were mounted with the ganglion layer side facing up, and coverslipped for optical microscopy. Vertical sections were mounted and coverslipped for examination under an optical microscope. Images were obtained on a high-resolution digital microscope (Leica DMR; Leica Microsystems) and processed using photo-editing software (Adobe Photoshop 10.0; Adobe Systems, Inc., San Jose, CA, USA).

A camera lucida connected to a Leica DMR microscope (Leica Microsystems) was used to trace by hand each immunostained mRGCs in all flat-mounted retinas examined in order to determine their type, number and spatial distribution. The resulting images were then digitalized, using image-editing software (Adobe Photoshop 10.0; Adobe Systems, Inc., San Jose, CA, USA).

### Morphological Analysis

Retinal flat mounts were photographed at the focal plane of the GCL and INL. In order to recreate the soma and dendritic profiles of the individual mRGCs, we traced by hand the shape of the cell body, the dendritic arbor and the minimal convex polygon that enclosed the dendritic field of several representative mRGCs (five cells per retina of each morphological subtypes found, 24 retinas analyzed) using a camera lucida. Measurements were taken of the areas of each soma and dendritic profile using ImageJ software (developed by Wayne Rasband from the National Institutes of Health, Bethesda, MD, USA and available at http://rsbweb.nih.gov/ij/index.html). Measurements of the cell body cross-sectional surface area were also taken in order to calculate the sizes of the somas, which are expressed as equivalent diameters (the diameter of a circle of equal area).

The Bonfire program from the Firestein laboratory at Rutgers University (Langhammer et al., 2010) was used to analyze the morphology of mRGC neurites. From digitized neuritic arbors, we estimated the number of branch points and terminal neurite tips per cell. A Sholl analysis (Sholl, 1953) was also conducted by drawing concentric circles with increasing radii around the cell body and counting how many times each circle crossed a neuritic segment. The number of intersections was plotted against the radial distance from the soma in order to obtain the Sholl profile and the Sholl area, i.e., the area under the profile (in arbitrary units).

The two-dimensional distribution of mRGCs was assessed by obtaining the Delaunay segments and the Voronoi domains associated with the mRGC mosaic using ImageJ software. The Voronoi domain area (VDA) of a cell is the area in the retinal plane whose points are all closer to said cell than to any other neighboring cells in the mosaic (Galli-Resta et al., 1997). Delaunay segments show the distance to the nearest neighbors (NND) of each cell. VDA and NND were plotted as histograms, fit to a Gaussian function and compared to the analysis of a random pattern, with the same density and standard deviation (SD; Noailles et al., 2014). Regularity index (RI) for each field were then calculated by dividing the mean NND or VDA by the SD (Raven et al., 2003; Reese and Keeley, 2015). There is a direct relationship between RI and the regularity of the mosaic; real mosaics yield nearest neighbors regularity indices (NNRI) greater than the index derived from a theoretical random distribution (1.91; Cook, 1996). The NND dispersion index (DI) was obtained by calculating the ratio between the observed mean NND and the mean distance of the nearest neighbor that can be obtained for the ideal random dispersion (Cook, 1996; Raven et al., 2003). The theoretical mean DI for a random distribution is 1. The higher the DI, the less similar the distribution is to a random pattern.

### Statistical Analysis

A two-way analysis of variance (ANOVA) was used to evaluate differences between mRGC subtypes (M1, M1d, M2 and M3) among the donors ages (<30, 30–50, 50–70 and >70 years of age), as well as the interactions among them, in terms of cell number, cell distribution pattern (RI and DI) and morphological parameters (soma diameter, dendritic area, terminal neurite tips, branch points and Sholl area). Normal homogeneity of variance and normal distribution were found for all analyzed categories. In cases where a 0.05 level of significance was found, *post hoc* pairwise comparisons by means of a Tukey’s test were conducted. Statistical significance was considered to be *p* < 0.05. Data were plotted as the mean ± SEM. A Grubb’s test was performed to determine significant outlier values. VDA and NND data were fitted to a Gaussian function. Prism 6 for Windows (Graphpad Software, Ind., La Jolla, CA, USA) was used for all statistical analyses.

## Results

### Melanopsin-Expressing Retinal Ganglion Cells in Human Retinas

**Figure 1:**
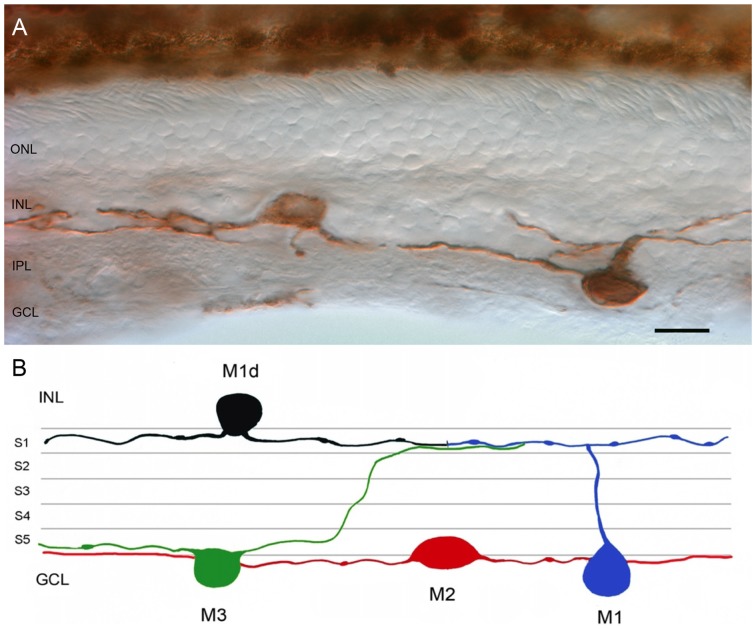
**Melanopsin-positive ganglion cells in the human retina. (A)** Representative image of a vertical section from a 53-year-old human retina labeled with anti-melanopsin antibody. Note that melanopsin is present on the soma and neurites of cells located in the GCL and INL of the human retina. **(B)** Representative drawing of the different types of melanopsin-expressing retinal ganglion cell (mRGC) found in human retina. ONL, outer nuclear layer; INL, inner nuclear layer; IPL, inner plexiform layer; GCL, ganglion cell layer. *Scale bar*: 20 μm.

Single immunolabeling of human vertical retinal sections, employing primary polyclonal antibody against human melanopsin in conjunction with an immunoperoxidase technique, was used to label positive ganglion cells. The expression of melanopsin was located in the bodies, axons and dendrites of some retinal ganglion cells. Melanopsin-positive cell bodies appeared in the GCL and inside the INL (Figure 1A). Dendritic processes were localized in two plexuses: one within the outer margin of the IPL, close to the INL (stratum S1 of the OFF sublamina), and the other on the inner side of the IPL, close to the GCL (stratum S5 of the ON sublamina).

### mRGC Types in the Human Retina

Figure 1B shows a schematic diagram of the different types of mRGCs found in human retinas. Based on the dendritic stratification patterns, we recognized three different subtypes of mRGCs in the human retina: M1 cells, with arborized dendrites in the S1 stratum of the IPL; M2 cells, stratifying in the S5 stratum of the IPL; and M3 cells, with bistratified dendrites in both S1 and S5 strata of the IPL. Weaker mRGC immunoreactivity was observed in M2 cells as compared to M1 and M3 cells. The cell bodies of M2 and M3 mRGC were located in the GCL. The cell bodies of numerous M1 cells were found in the GCL, but the cell bodies of most mRGCs stratifying in S1 were displaced to the INL. As a result, they are understood to be displaced M1 cells (M1d; Figures 1A,B). To our knowledge, this is the first work to describe M3 cells in the human retina.

**Figure 2:**
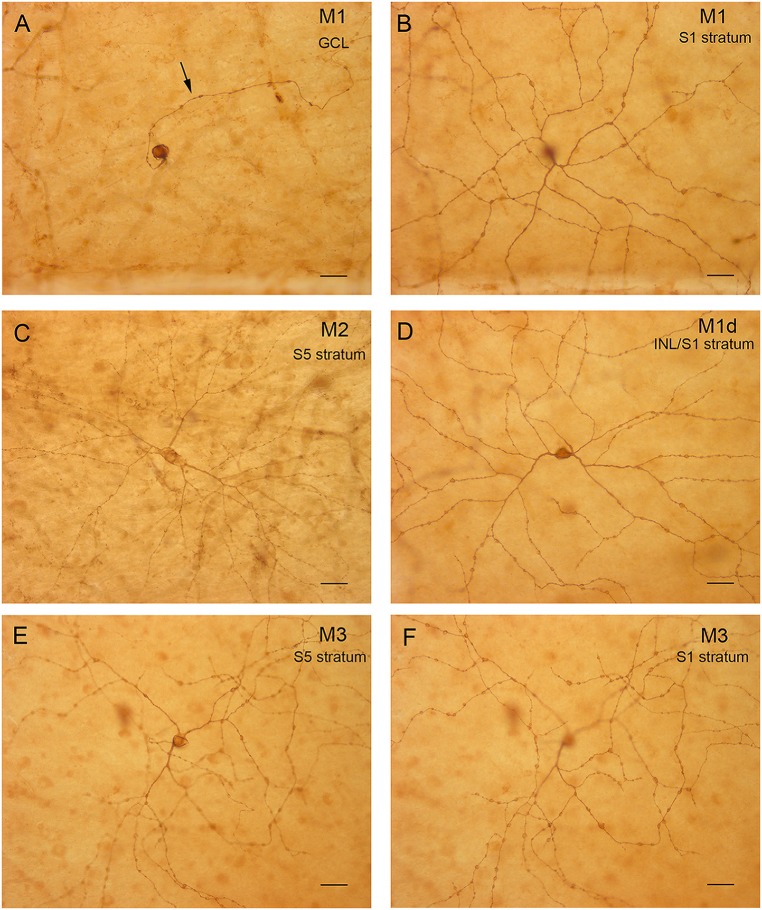
**mRGC types in the human retina. (A,B)** Representative M1 cell with the soma and axon (arrow) located in the GCL **(A)** and dendrites in the stratum S1 of the IPL **(B)**. **(C)** M2 cell with the cell body located in the GCL and dendrites in S5 of the IPL. **(D)** Displaced M1 cell (M1d) with the body located in the INL and dendrites in the stratum S1 of the IPL. **(E,F)** M3 cell with the cell body located in the GCL **(E)** and dendrites in both S5 **(E)** and S1 of the IPL **(F)**. *Scale bar*: 40 μm.

**Figure 3:**
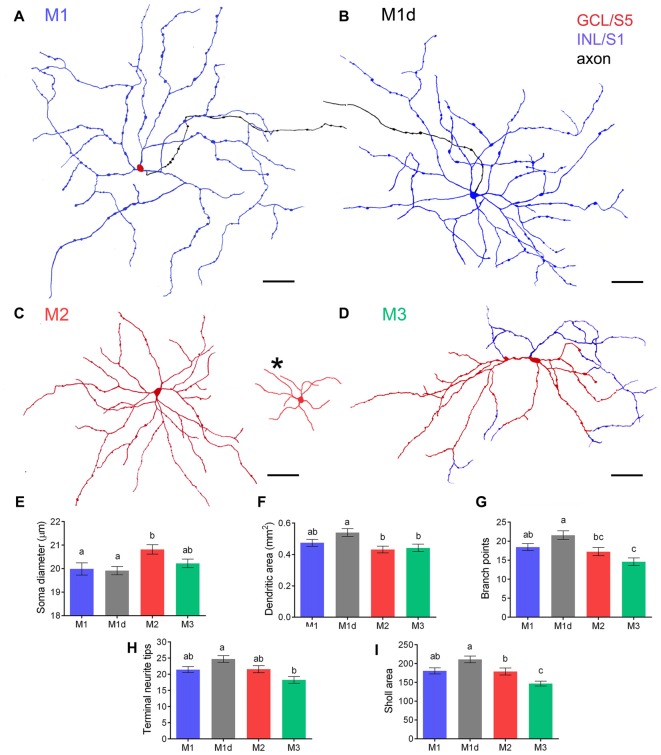
**Dendritic arborization of mRGCs in the human retina. (A–D)** Examples of soma-dendritic profiles of M1, M1d, M2 and M3 cells. Dendrites in stratum S1 and S5 are shown in *blue* and *red*, respectively, whereas axons are illustrated in *black*. Somas located in the GCL (M1, M2 and M3 cells) and INL (M1d cells) are shown in *red* and *blue*, respectively. A group of smaller M2 cells was found close to the optic nerve (**C**, asterisk). **(E)** Mean soma diameter of M1, M1d, M2 and M3 cells. A total of 400 cells were examined for each cell type. **(F–I)** Mean dendritic area **(F)**, number of branch points **(G)**, number of terminal neurite tips per cell **(H)**, and Sholl area **(I)** of each mRGC type. 40–45 cells were examined for each cell type. Different letters indicate statistical significance. *Scale bar:* 100 μm.

To analyze the stratification pattern and morphology of mRGCs in detail, retinal flat mounts were immunostained for melanopsin using the immunoperoxidase technique (Figure 2), and individual cells were drawn by hand, using a camera lucida (Figures 3A–D). Soma size and neurite morphology were analyzed. In all mRGC types, soma profiles were circular in shape (Figures 2, 3), with soma diameters significantly larger in M2 (20.82 ± 0.19 μm) than in M1 (19.99 ± 0.26 μm) and M1d cells (19.92 ± 0.17 μm; *p* < 0.05 and *p* < 0.01, respectively; Figure 3E). M3 cells had somas with diameter values (20.22 ± 0.18 μm) that fell between those observed in M1 and M2 cells. All cell types showed branched dendritic trees of beaded dendrites (Figures 2, 3). The mean dendritic area of M1d cells (0.54 ± 0.02 mm^2^) was higher than that of M2 (0.43 ± 0.02 mm^2^) and M3 (0.44 ± 0.02 mm^2^) cells (*p* < 0.01 and *p* < 0.05, respectively; Figure 3F). No significant differences in the dendritic area of M1 cells (0.47 ± 0.02 mm^2^) were found in any of the other cell types. The Bonfire analysis also showed significant differences in dendritic tree morphology among the different cell types in human retinas. With regard to the quantity of branch points per cell (Figure 3G), both M1 (18.43 ± 0.92) and M1d (21.57 ± 1.16) cells appeared in greater numbers than M3 cells (14.58 ± 1.02; *p* < 0.05, in both cases), and M2 cells showed lower values (17.24 ± 1.06; *p* < 0.05) than M1d cells, but were not significantly different from those observed in M1 and M3 cells. The number of terminal neurite tips per cell was also significantly larger in M1d (24.75 ± 1.05) than in M3 cells (18.28 ± 1.06; *p* < 0.001), although they did not show any significant differences from M1 (21.44 ± 0.93) and M2 cells (21.59 ± 1.09; Figure 3H). The Sholl area was larger in M1d (211.1 ± 8.9) than in M2 (179.0 ± 9.3) and M3 (146.7 ± 6.5) cells (*p* < 0.05 and *p* < 0.0001, respectively), and M1 cells showed Sholl area values (180.7 ± 8.05) that were between those observed in M1d cells and M2 cells (Figure 3I). All these results indicate that M1d cells had a more complex dendritic arborization than the other mRGC types, and that M3 cells had the simplest dendritic trees among the different mRGC morphologies. Close to the optic nerve, we found a few mRGCs with somas placed in the GCL and small dendritic trees with short process stratifying in the S5 stratum of the IPL. The immunoreactivity of these small M2 cells was weaker than in the rest of M2 cells (Figure 3C, asterisk).

### mRGC Distribution and Number in Human Retinas

**Figure 4:**
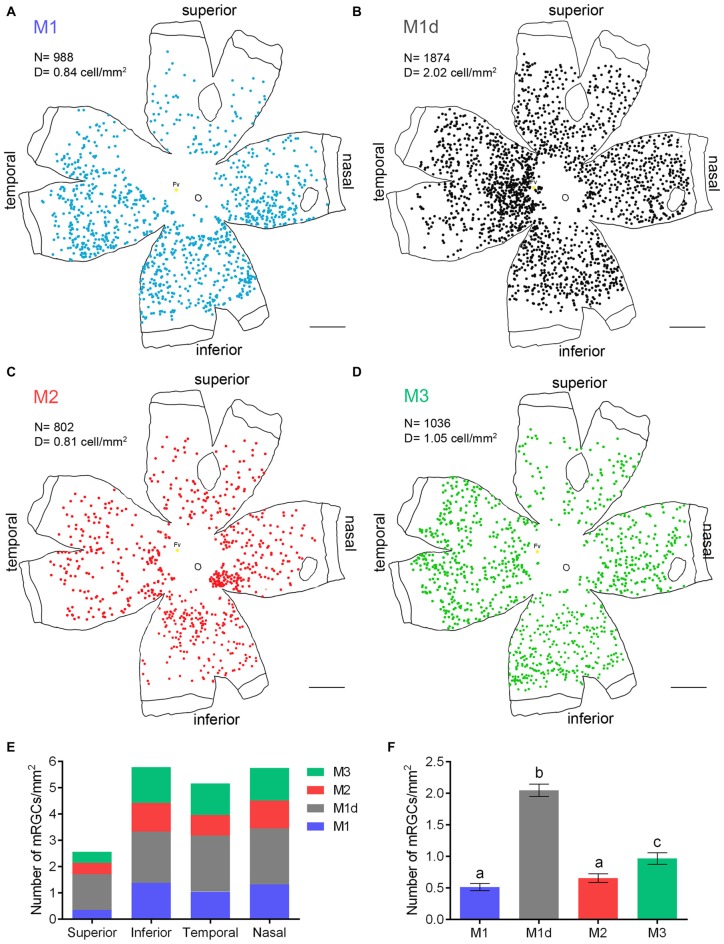
**Density and distribution of the different types of human mRGCs. (A–D)** Representative drawings of a 56-year-old whole-mount human retina (drawn by hand, using a camera lucida) showing the location of immunostained individual mRGCs of different types. The number of mRGCs shown in the drawings is 988 M1 **(A)**, 1874 M1d **(B)**, 802 M2 **(C)** and 1036 M3 **(D)** cells. **(E)** Density of the different types of mRGCs (cell/mm^2^) in different parts of the whole mount retina showed in **(A–D)**. **(F)** Mean density of the different types of mRGCs (cell/mm^2^) in the superior-nasal part of the retina of 24 human donors aged 10–81 years. Fv, fovea; N, number of mRGCs; D, density of cells. Different letters above the histograms indicate statistical significant differences (*p* < 0.05). *Scale bar*: 5 mm.

In order to gain a complete perspective of the spatial distribution and number of mRGCs in the human retina, each and every individual mRGC from an entire flat mounted human retina from a 56-year-old donor was morphologically characterized and the cell body manually traced using a camera lucida. Figure 4 presents the spatial arrangement of the different types of mRGCs in the whole-mount human retina analyzed (Figures 4A–D). Throughout the entire retina, the total number of mRGCs was 4700, and the average cell density was 4.77 cells per mm^2^. A higher density of mRGCs was observed around the fovea, and a reduced density of mRGCs was found in the vicinity of the optic nerve and close to the peripheral retina. The smallest density for all mRGCs types was observed in the superior part of the retina (Figure 4E). On the other hand, we observed a high proportion of M1d cells throughout the entire retina.

To obtain an average estimation of the relative density of each mRGC-type population, each mRGC-type was counted in a representative region of around 1 cm^2^ from the superior-nasal area of 24 retinas from donors aged 10–81 years. The mean density of M1d cells in this part of the retina (2.05 ± 0.47 cells/mm^2^) was significantly greater than that of M1 (0.51 ± 0.27 cells/mm^2^), M2 (0.65 ± 0.33 cells/mm^2^) and M3 cells (0.97 ± 0.44 cells/mm^2^; *p* < 0.001;* n* = 24 in all cases). The mean density of M1 and M2 cells was not significantly different, and M3 cell density was significantly higher than that measured in M1 and M2 cells (*p* < 0.001 and *p* < 0.05, respectively;* n* = 24; Figure 4F).

**Figure 5:**
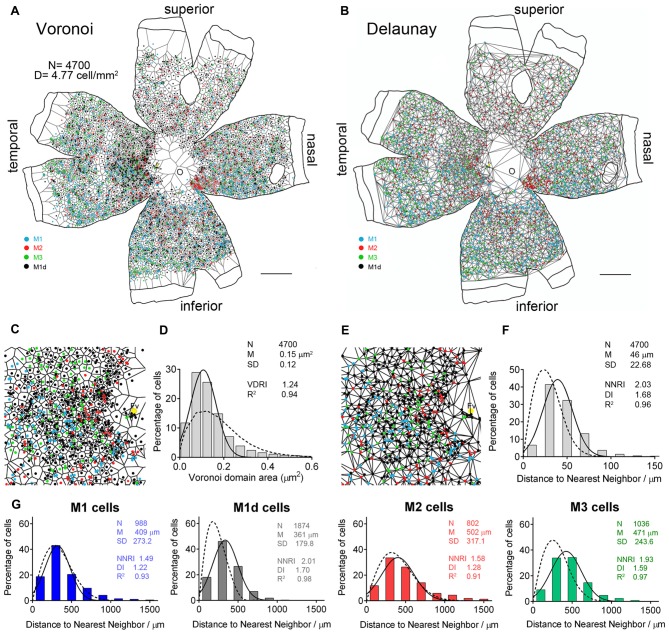
**Distribution pattern of human mRGCs. (A)** Voronoi tessellation associated with the mRGC mosaic of a 56-year-old whole human retina. **(B)** Delaunay tessellation associated with the mRGC whole retina mosaic, as well as the set of Delaunay segments for single cells, the shortest of which is the distance of the nearest neighbor to each cell. **(C)** Higher magnification image of Voronoi tessellation. **(D)** Frequency distribution associated with the Voronoi domains, showing the regularity index (VDRI). **(E)** Higher magnification image of Delaunay tessellation close to the fovea area (Fv). **(F)** Frequency distribution of the nearest neighbor densities of mRGCs, independently of the cell type, showing the nearest neighbor regularity index (NNRI) and dispersion index (DI). The solid line represents the Gaussian function fitting the data. The Voronoi **(D)** and Delaunay **(F)** analysis of a random pattern with the same density and SD is also shown for the purposes of comparison (dotted line). **(G)** Frequency distribution of NND for the different types of mRGCs in the whole-mount retina showed in **(A,B)**. N, number of cells; M, mean value (area or distance); SD, standard deviation; *R*^2^, R-squared value. *Scale bar*: 5 mm.

To better characterize the two-dimensional distribution of mRGCs, we analyzed the Voronoi domains and the Delaunay segments of melanopsin-positive cells throughout the entire flat mounted retina from a 56-year-old donor. Examples of the resulting Voronoi and Delaunay tessellations, as well as the histograms generated from these analyses, are shown in Figure 5. The Voronoi analysis for all stained cells, independently of the cell type, revealed that mRGCs follow a Gaussian distribution (*R*^2^ = 0.94) in the human retina (Figure 5D, solid line). However, the VDA histogram was similar to that described by a Voronoi analysis of a random pattern with the same density and SD (Figure 5D, dotted line), and Voronoi domains, showing the regularity index (VDRI) reached a relatively low value (1.24). In contrast, the Delaunay analysis showed that NND histograms for all mRGCs, independently of the cell type, were well-fitted by a Gaussian function (*R*^2^ = 0.96; Figure 5F, solid line), and a Delaunay analysis of a random pattern could not describe these histograms (Figure 5F, dotted line). Moreover, NNRI and DI reached relatively high values (2.03 and 1.68, respectively), indicating that, taken together, all mRGCs are arranged in a fairly regular mosaic.

The Delaunay analysis for each of the mRGCs types in the entire flat-mounted human retina showed regularity and dispersion indices that were relatively high for M1d (2.01 and 1.70, respectively) and M3 (1.93 and 1.59, respectively) cells. Moreover, Delaunay analyses of random patterns of the same density and SD could not describe NND histograms for these cell types (Figure 5G, dotted lines), indicating that the NND are uniform, and that M1d and M3 cells are organized into a fairly regular mosaic. In contrast, regularity and dispersion indices for M1 (1.49 and 1.22, respectively) and M2 (1.58 and 1.28, respectively) cells were relatively low, and NND histograms for these cell types were well-fitted by the Delaunay analysis of random patterns (Figure 5G), indicating that retinal distribution of M1 and M2 cells was close to a random pattern.

### Decrease and Degeneration of mRGCs in the Human Retina with Aging

**Figure 6:**
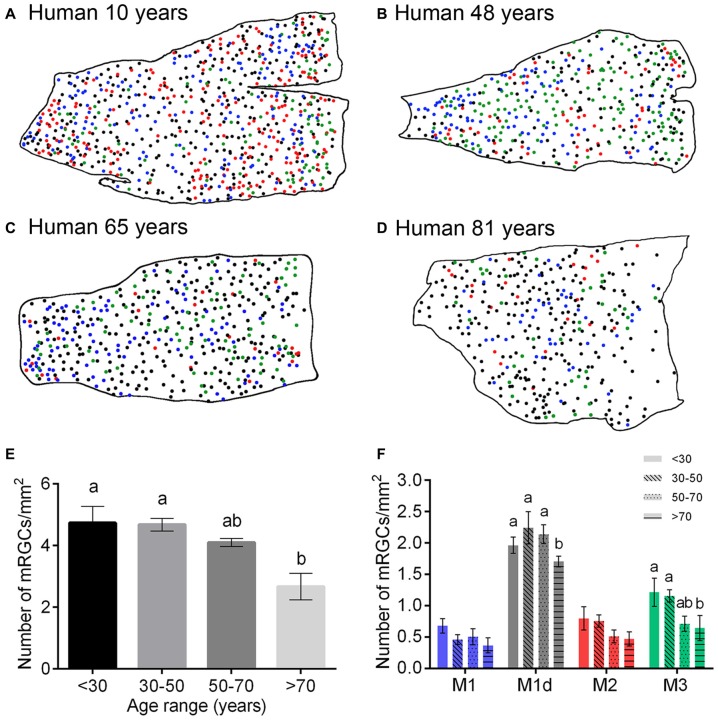
**Number of mRGCs in the human retina with aging. (A–D)** Representative drawings of the superior-nasal part of whole-mount retinas from humans at 10 **(A)**, 48 **(B)**, 65 **(C)** and 81 **(D)** years of age, corresponding to the age ranges: <30, 30–50, 50–70 and >70 years. **(E,F)** Mean density of total mRGCs **(E)** and different types of mRGCs **(F)** in human retinas between 10 and 81 years of age. Note the significant decrease in the number of mRGCs as of 70 years of age. Six retinas were examined for each age range. Different letters above the histograms indicate statistical significant differences (*p* < 0.05). *Scale bar*: 1 mm.

To assess age-dependent variations in mRGC numbers, distribution and/or morphology, a representative region of around 1 cm^2^ from the superior-nasal area of 24 flat mount human retinas of different ages (10–81 years) was analyzed. To calculate the relative quantities of each cell type in each human retina, each of the cell bodies stained for melanopsin was traced by hand, using a camera lucida (Figures 6A–D). The mean melanopsin immunoreactive cell density showed a decrease throughout the course of life in human retinas, with significant differences between humans less than 70 years of age and humans older than 70 years (*p* < 0.01, *n* = 6 in all age ranges; Figure 6E). This age-dependent decrease in mRGCs was found in all the cellular types of mRGCs, with significant differences in M1d and M3 cells (Figure 6F).

**Figure 7:**
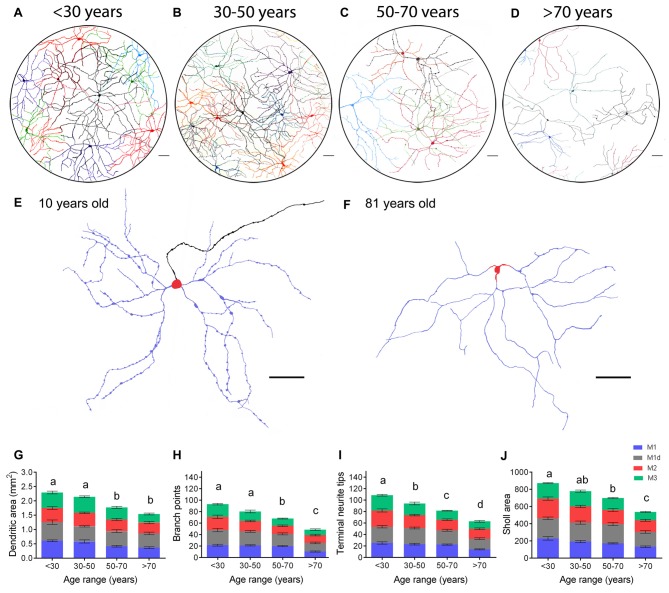
**mRGC morphological and arborization changes in human retinas with aging. (A–D)** Representative drawings of the soma and complete dendritic field of mRGCs from the central superior-nasal retinal region of human retinas at 10 **(A)**, 46 **(B)**, 53 **(C)** and 89 **(D)** years of age, corresponding to the age ranges: <30, 30–50, 50–70 and >70 years. **(E,F)** Examples of dendritic profiles of M1 cells from human retinas at 10 **(E)** and 81 **(F)** years of age. Dendrites, in stratum S1, are shown in *blue*. Somas, located in the GCL, are shown in *red*. Axon is illustrated in *black*. **(G–J)** Average dendritic area **(G)**, number of branch points **(H)**, number of terminal neurite tips per cell **(I)**, and Sholl area **(J)** of the different types of mRGCs in human retinas with ages belonging to the ranges: <30, 30–50, 50–70 and >70 years. 12–17 cells from nine retinas were examined per age range. Different letters above the histograms indicate statistical significant differences (*p* < 0.05). *Scale bar*: 100 μm.

To analyze age-dependent morphologic changes in mRGCs, we manually traced the cell body and the dendritic field of representative mRGCs from the 24 retinas analyzed (five cells per retina of each morphological subtype described). Figure 7 presents the soma reconstruction and the full dendritic field of individual mRGCs in a representative region of the central superior-nasal area of representative retinas aged <30, 30–50, 50–70 and >70 years (Figures 7A–D). As shown in the figure, there was extensive overlapping of the dendritic fields of neighboring mRGCs, forming a dense meshwork in the IPL in retinas younger than 30 years of age (Figure 7A). The dendritic fields were organized in a similar manner in retinas from 30 to 50-year-old donors (Figure 7B). From 50 years of age onward, it was observed that the mRGC plexus was less complex than in younger specimens, the dendritic fields overlapped to some degree, but with visible empty spaces in coverage (Figure 7C). Over age 70, the mRGCs were few and far between, with barely any contact between cells (Figure 7D). The dendritic beads observed in all types of mRGCs (Figures 2, 3, 7E) were lost with age. Compare representative M1 cell at 10 year old (Figure 7E) with that at 80 year old (Figure 7F).

The morphologic parameters quantified in mRGCs confirmed the age-related reduction in dendritic area, number of terminal neurite tips per cell, number of branch points, and Sholl area in the human retinas (Figures 7G–J). More specifically, between <30 and 30–50 years of age, we already found a significant decrease in terminal neurite tip numbers (*p* < 0.05). In addition, from 50 years of age onward, a significant decrease was observed in all the dendritic parameters analyzed, with regard to measurements in <30-year-old retinas (*p* < 0.0001, in all cases). From 70 years of age onwards, the value of the Bonfire dendritic parameters analyzed were also significantly smaller than those obtained in humans in the 50–70 year-old range (*p* < 0.001, in all cases; Figures 7H–J).

**Figure 8:**
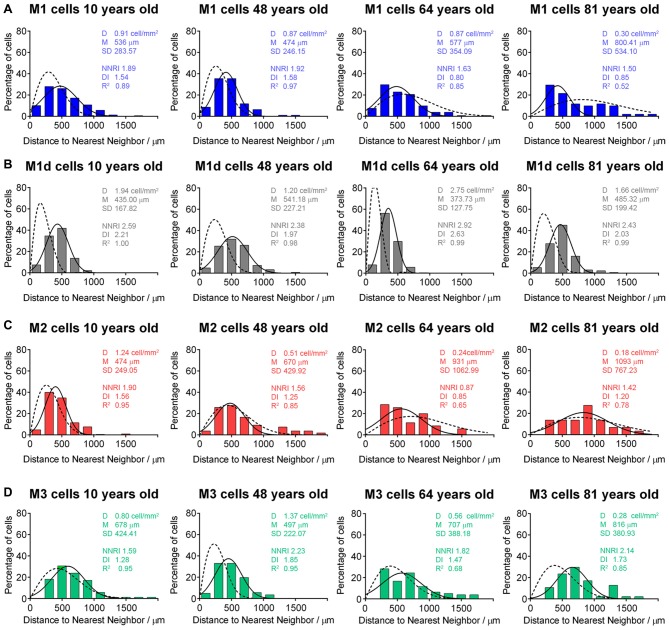
**Distribution pattern of mRGCs in human retinas with aging. (A–D)** Frequency distribution of the nearest neighbor densities of M1 **(A)**, M1d **(B)**, M2 **(C)** and M3 **(D)** cells in four representative human retinas at 10, 48, 65 and 81 years of age, corresponding to the age ranges: <30, 30–50, 50–70 and >70 years. The solid line in each histogram represents the Gaussian function fitting the data. The dotted line corresponds to the Voronoi analysis of a random pattern with the same density and SD. D, mean density; M, mean distance; SD, standard deviation; NNRI, nearest neighbor regularity index; DI, dispersion index; *R*^2^, R-squared value.

Age-related changes in the mRGC distribution were assessed by analyzing the Delaunay segments of melanopsin-positive cells in a representative region (1 cm^2^, superior-nasal area) of four flat-mounted human retinas of different ages (<30, 30–50, 50–70 and >70 years of age). Histograms showing NND values from these analyses are presented in Figure 8. As shown in the figure, the distribution pattern of M1 and M2 cells grew progressively closer to a random pattern with aging (Figures 8A,C). More concretely, the fit of NND values in M1 and M2 cells to a Gaussian function (Figure 8, solid lines) grew progressively closer to the NND values of random patterns having an identical density and SD (Figure 8, dotted lines), and regularity and dispersion indices were progressively lower with aging. These cell types were arranged in a fairly regular mosaic at young ages (RI around 1.91), but with age they showed a more random distribution. Nevertheless, M3 cells were arranged in a fairly regular mosaic (NNRI) approximating the theoretical limit of 1.91 for a random population (Cook, 1996) in most of the examined ages (Figure 8D). M1d cells were arranged in a regular mosaic (NNRI > 1.91) in all of the ages examined (Figure 8B).

## Discussion

The present study shows that human retinas contain a minimum of three melanopsin-positive retinal ganglion cells subtypes (M1, M2 and M3), distributed throughout the entire retina in cell mosaics. These are more or less regular, depending on the cell subtype. Here we also demonstrate that mRGC density significantly decreases in people over 70 years of age, even though a progressive decline is observed from age 50, accompanied by a reduction in dendritic arborization in association with age. Over the course of a lifetime, the distribution profile of mRGCs grows progressively closer to a random pattern.

Over the entire human retina, immunolabeling with melanopsin antibodies revealed a morphologically distinct population of 4700 cells (4.77 cells/mm^2^), which correspond to 0.4% of the entire population of ganglion cells in the retina (assuming that the human retina has a total of 1.07 million RGCs; Curcio and Allen, 1990). This value is comparable to those reported by Liao et al. (2016). In other previous studies, however, the reported density of human mRGCs was between ≈0.8% (Hannibal et al., 2004) and 1.5% (La Morgia et al., 2010) of the total number of ganglion cells. These differences could be due to the fact that, in these studies, the analyses were conducted in randomly selected areas (not throughout the entire retina), to differences in the labeling methods, or both. An increased density of mRGCs was found in the temporal part of the retina, close to the parafoveal retina, which is in accordance with previously reported data (Dacey et al., 2005; La Morgia et al., 2010; Liao et al., 2016). Furthermore, in the far periphery, we found a greater concentration of mRGCs within the nasal retina. In agreement with this result, a peak in the quantity of mRGCs on the edge of the nasal hemiretina has been previously reported (La Morgia et al., 2010). The distribution patterns of mRGCs previously reported for non-human species (Hattar et al., 2002; Semo et al., 2005; Esquiva et al., 2013) are different from those found in humans.

### Distribution and Types of mRGCs in Human Retinas

Two morphologically distinct melanopsin-positive cells have been previously reported in three species of primates, namely marmosets (Jusuf et al., 2007), macaques and humans (Hannibal et al., 2004; Dacey et al., 2005). In one case, the dendrites were monostratified in the outermost stratum of the IPL, whereas the dendrites of the second type were located mainly in the innermost stratum. This anatomical description of primate melanopsin cells, which are similar to rodent M1 and M2 cells, was later confirmed (Dkhissi-Benyahya et al., 2006; La Morgia et al., 2010; Neumann et al., 2011) and the cells have been referred to accordingly (Neumann et al., 2011).

Immunoperoxidase studies enabled us to clearly classify human mRGCs into three different cell subtypes: M1, M2 and M3. M1 cells, with dendrites stratifying exclusively in the outer IPL, constituted 12% of the total mRGCs. Cells with the same characteristics were previously found in primates (Dacey et al., 2005; Jusuf et al., 2007). A population of mRGCs stratifying in the IPL’s outer stratum had their soma displaced in the INL, and thus were considered as displaced M1 cells. Other researchers have previously described mRGCs with the same morphology in the retina of rats (Engelund et al., 2010; Esquiva et al., 2013, 2016), mice (Schmidt and Kofuji, 2009; Berson et al., 2010) and humans (Hannibal et al., 2004; Dacey et al., 2005; La Morgia et al., 2010). As occurs in the rat, M1d cells present a great similarity to M1 cells with regard to dendritic area, morphology and distribution of its dendrites, and for that reason we consider them to fall within the M1 group. Jusuf et al. (2007) reached this same conclusion when they found that some cells stratifying into the outer layer of the IPL could have their soma displaced to the INL in the retina of the common marmoset (Jusuf et al., 2007). M1d cells were the predominant cell subtype in the human retinas (49%), corroborating previous studies in primates (Hannibal et al., 2004; Dacey et al., 2005; Jusuf et al., 2007). The proportion of M1 and M1d represented about 60% of the total mRGCs, which is in accordance with what has been previously reported by Dacey et al. (2005) for outer-stratifying cells in the retinas of macaques and humans.

The dendrites of the second cell type we found, M2, project only to the inner melanopsin-immunoreactive plexus, remaining close to the GCL, in the stratum S5 of the IPL. These cells probably correspond to previously documented mono-stratified melanopsin cells in human and non-human primates (Dacey et al., 2005; Jusuf et al., 2007), whose dendrites project to the innermost IPL layer (S5). The proportion of these cells was similar to the proportion of M1 cells (16%). In addition, close to the optic nerve, a group of M2 cells was found with small dendritic trees. Small mRGCs has been previously described in macaques (Liao et al., 2016).

We found a third mRGCs cell type, M3, which had not been previously described in humans and whose dendrites projected in both strata of the IPL. In most cases, the dendrites of these bistratified cells were divided almost evenly between each stratum, but there were also cells with a majority of their dendrites in the outer stratum and cells whose dendrites were mostly located in the inner stratum. In marmoset (Jusuf et al., 2007), human and macaque (Liao et al., 2016) retinas, both M1 and M2 cells projecting some of their processes into the “wrong” plexus have been occasionally seen, but no bistratified cells had been reported so far in humans. Bistratified or M3 mRGCs with dendrites projecting in both IPL strata have been previously reported in mice and rats (Viney et al., 2007; Schmidt et al., 2008; Berson et al., 2010; Esquiva et al., 2013). Some authors argued that bistratified mRGCs in mice cannot be considered a distinct cell type because they do not form a distinct mosaic that uniformly tiles the retina (Berson et al., 2010). However, we found M3 cells regularly distributed throughout the entire human retina. Nevertheless, it will be necessary to conduct electrophysiological experiments to ascertain whether human melanopsin bistratified cells have characteristic physiological properties that are similar to those reported for M3 cells in mice (Schmidt and Kofuji, 2011). In rodents, the number of bistratified mRGCs was fewer (approximately 10%; Berson et al., 2010; Esquiva et al., 2013) as compared to what we found in human retinas (23%). In the present study we identified, for the first time, a bistratified subtype of mRGCs in human retinas, showing that the human retina contains at least three different subtypes of mRGCs (M1–M3). The dendrites of all these types of mRGCs form a dense meshwork, as previously described (Provencio et al., 2002; Jusuf et al., 2007).

Regarding the staining intensity of these cell types, M2 cell immunoreactivity was weaker than that of M1 and M3 human cells, which agrees with previous studies on mice (Berson et al., 2010) and rats (Esquiva et al., 2013). In humans, there are two isoforms of Opn4 (Jagannath et al., 2015). Thus, the low immunoreactivity of M2 cells may be due to the fact that, contrary to what occurs in other cell types, only one of the isoforms is expressed in these cells, as previously described in mice (Pires et al., 2010). Another possibility is that both isoforms are expressed in all three cell subtypes, but the concentration of the protein is substantially lower in M2 than in M1 and M3 cells.

Having several different types of mRGCs appears to be a common feature among mammals. In mouse (Ecker et al., 2010) and rat (Reifler et al., 2015) retinas, two other subtypes of ipRGCs (M4 and M5) have been described. However, M5 cells are melanopsin immunonegative in both animal species, and M4 cells show melanopsin immunostaining in mice only after strong amplification of the immunofluorescence (Estevez et al., 2012) and they lack any obvious staining in rat retinas (Esquiva et al., 2013; Reifler et al., 2015). These results suggest that the lowest expression levels among ipRGCs are found in M4 and M5 cells, with levels below the detection threshold either because of the staining method or the presence of a photopigment other than melanopsin. Thus, it is entirely possible that human retinas have more types of ipRGCs than were found in our study. Once again, electrophysiological studies would be needed to determine whether M4 and M5 cell types exist in the retina of humans.

In our results, the distribution of M1d and M3 cells form fairly regular cellular mosaics. Conversely, M1 and M2 cells are arranged in more irregular mosaics within the GCL. It may be that M1 and M2 cells are not arranged according to a regular distribution due to their low density. Raven et al. (2003) have found that low-density cell mosaics can resemble random distributions. Retinal cells that share a common function are usually regularly distributed. However, it has been reported that in cat retinas, mRGCs have a random distribution throughout the GCL (Semo et al., 2005).

### Age-Dependent Changes in Density, Distribution and Morphology of Human mRGCs

Previous studies in humans showed that between 31 and 60 years of age, the axon population of retinal ganglion cells progressively decreases (Johnson et al., 1987). Since then, different researchers have found that the number of RGC in the GCL decreases with age (Gao and Hollyfield, 1992; Curcio and Drucker, 1993; Harman et al., 2000). The loss of RGC reported for the entire human retina is 0.5% per year, with a total loss of 38% over the course of a lifetime (Harman et al., 2000). On the other hand, evidences suggest a progressive increase in the retinal area in humans until approximately 30 years of age, remaining relatively constant for the rest of the life (Harman et al., 1997). Furthermore, resistance of mRGCs to cell injury has been reported in different animal models (Robinson and Madison, 2004; Li et al., 2006, 2008). In humans, it has also been shown that mRGCs resist neurodegeneration as the result of mitochondrial dysfunction, corroborating the robustness and resistance of these cells (La Morgia et al., 2010).

In this work, we show that human mRGC density is not strongly affected until advanced ages, with no significant differences being found until 70 years of age. From 50 years of age onward, we observed a tendency to decrease, which amounted to just about 13%, a lower value than that reported for total RGCs (about 30%; Harman et al., 2000). However, a marked drop in mRGCs was seen after age 70, with a significant decrease of approximately 44% in the total number of mRGCs (as compared to the youngest retinas). Since the size of the retina remains relatively constant from the age of 30 in humans (Harman et al., 1997), this fall in the mRGC density from 70 years old is not attributable to changes in the retinal size. On the other hand, dendritic parameters indicated a progressive atrophy of mRGC dendritic trees with aging. The atrophy of the dendritic trees start before mRGCs number decreasing. After 50 years of age, mRGC plexuses were significantly less complex, and from 70 years of age onward, mRGCs showed little synaptic contacts and overlapping of dendritic fields between the scarce number of remaining cells. Moreover, after 70 years of age, the distribution profile of mRGCs was closer to a random pattern than that observed in younger retinas.

All these results are in concordance with previous studies showing age-related decreases of mRGCs in animal models (Semo et al., 2003; Esquiva et al., 2013; Lax et al., 2016). Furthermore, La Morgia et al. (2010) compared post-mortem retinal specimens from two donors in their mid-fifties and another over 80 years of age, and found that mRGCs are lost with age. In humans, the presence of lipofuscin deposits have also been reported in melanopsin cells, something that could potentially have a progressive impact on their functionality with age (Vugler et al., 2007). The progressive age-dependent loss of mRGCs can explain the fact that in older retinas, the distribution profile of mRGCs was closer to a random pattern than in younger retinas. In this sense, it has been reported that cell mosaics with low densities can resemble random distributions (Raven et al., 2003). The advanced age at which we found a significant decrease in mRGC density agrees with previous studies revealing the resistance of mRGCs to cell injury (Robinson and Madison, 2004; Li et al., 2006, 2008; La Morgia et al., 2010). Moreover, the recently described lack of effect of age on M1 cell inputs to the human pupil control pathway prior to 70 years of age (Adhikari et al., 2015) is also in concordance with our results, which reveal no significant differences in the number of M1 cells before this age.

Age-related numerical and morphological changes in human mRGCs occurred in all cell types. This fact is interesting because different types of melanopsin-expressing cells project to different areas of the brain, and are therefore involved in different functions, including the photosynchronization of the biological clock, sleep regulation, pupillary constriction, cognition etc. Although we only found statistically significant differences in M1d and M3 cells, the other two mRGC types showed a progressive and more regular decline. Because we don’t have data about the specific function of M1d, M2 and M3 melanopsin cell types, it is difficult to assign which cell type is involved in the impairment of the circadian rhythms in humans. Thus, the loss of all types of melanopsin cells can affect all the functions in which these photoreceptors are involved. The marked decline in mRGC density after 70 years of age in human retinas may therefore be the cause of the circadian rhythm desynchronization observed in elderly humans (Myers and Badia, 1995; Tales et al., 2001; Cajochen et al., 2006; Schmidt et al., 2012). Other alterations of the circadian cycles also occur with age, such as the gradual decrease in nocturnal melatonin secretion (Karasek and Reiter, 2002) and alterations in the sleep/wake phases (Neikrug and Ancoli-Israel, 2010), the functioning of which involves ipRGCs. All these alterations have been considered within the wide variety of pathologies associated with age (Gibson et al., 2008). It has recently been shown that variations in the melanopsin phototransduction circuitry can partially account for individual differences in sleep timing (van der Meijden et al., 2016). In glaucoma, that course with ganglion cell death, a loss of circadian rhythms and impairment of pupillary constriction have been reported (Feigl et al., 2011; Kankipati et al., 2011; Nissen et al., 2014; Obara et al., 2016) that could be related to the loss of mRGCs. Also, the PLR is reported to be reduced in amplitude in Alzheimer’s disease (Tales et al., 2001; La Morgia et al., 2016) that could be correlated with the loss of mRGCs in this disease and circadian dysfunction (Feng et al., 2016).

Circadian rhythms and other functions in which mRGCs are involved can be affected by both the atrophy of the dendritic trees and the loss of mRGCs. In our results the dendritic impairment reduces the number of synaptic contacts between mRGCs, which may contribute to circadian rhythm desynchronization. On the other hand, the age-related reduction in dendritic arborization could result in decreased amounts of available photopigment and, therefore, in reduced light sensitivity. In advanced stages of retinal degeneration, previous studies in rats provide evidences that the progressive loss in both density and dendritic arborization of mRGCs correlates with the occurrence of circadian dysfunctions (Lax et al., 2016).

We can conclude that advanced age is associated with a progressive loss in the density and dendritic arborization of the three subtypes of mRGCs found in human retinas, possibly accounting for the more frequent occurrence of circadian rhythm disorders in elderly persons, including sleep disturbances and cognitive alterations, such as learning, memory and mood. All this changes can be a consequence of the normal process of aging/senescence of the retina as well as other brain areas. Preservation of human retinal degenerations can prevent circadian disorders in older humans. Therefore, these results highlight the importance of protecting and taking care of the retina, and melanopsin cells, throughout the entire lifetime.

## Author Contributions

NC was in charge of the experimental design. GE conducted the experiments, acquired and analyzed the data and drafted the manuscript. PL helped analyze the data and revise the manuscript. GE, PL, JMG-F, JJP-S and NC discussed the results and manuscript.

## Funding

This research was supported by grants from the Spanish Ministry of Economy and Competitiveness (MINECO-FEDER BFU2015-67139-R), Instituto de Salud Carlos III (RETICS-FEDER RD16/0008/0016) and Generalitat Valenciana (PROMETEO/2016/158).

## Conflict of Interest Statement

The authors declare that the research was conducted in the absence of any commercial or financial relationships that could be construed as a potential conflict of interest.
